# A modern web-based health promotion program for patients in Greece with diabetes 2 and obesity: an interventional study

**DOI:** 10.1186/s12889-023-15557-3

**Published:** 2023-04-03

**Authors:** Maria S. Chrysi, Ioannis Michopoulos, George Dimitriadis, Melpomeni Peppa

**Affiliations:** 1 Intensive Care Unit, Hellenic Anticancer Institute, “Saint Savvas” Hospital, Athens, Greece; 2 2nd Department of Psychiatry, Attikon University Hospital, National and Kapodistrian Athens University Medical School, Athens, 12462 Greece; 3 2nd Propaedeutic Department of Internal Medicine, Attikon University Hospital, National and Kapodistrian Athens University Medical School, Athens, 12462 Greece; 4 Endocrine Unit, 2nd Propaedeutic Department of Internal Medicine, Attikon Univeristy Hospital, National and Kapodistrian Athens University Medical School, Athens, Greece

**Keywords:** Health promotion, Obesity, Diabetes, Interactive website

## Abstract

**Background:**

Health promotion programs are most beneficial in chronic diseases such as diabetes and morbid obesity, which can be positively affected by changes in attitudes, beliefs, and lifestyle.

**Objectives:**

This study aimed to develop an internet-based modern Health Promotion model using interactive online applications through continuing education and participation.

**Methods:**

The goal was to positively impact knowledge, behavior, and quality of life for patients with obesity and/or diabetes. This is a prospective interventional study on patients with obesity or type 2 diabetes. Seventeen two patients who met the inclusion criteria were distributed randomly into two groups (control and intervention) from 2019 to 2021 in Greece. All the participants were given questionaries concerning quality of life anxiety and depression (HADS) attitudes and beliefs, knowledge about their condition and general questions to establish a baseline. A traditional health promotion model was followed for the control group. For participants in the intervention group, a web-based health promotion program was created according to the goals of the research. Participants were instructed to log on 1–2 times a week for 5–15 min, with the understanding that the research team would be monitoring their activities. The website included two knowledge games and personalized educational material based on their needs.

**Results:**

The sample comprised 72 patients (36 in control and 36 in the intervention groups). The mean age was 47.8 years for the control group and 42.7 years for the intervention group (p = 0.293). Both study groups had a significant increase in knowledge score on diabetes (Control group:3,24, Intervention group 11,88 p < 0,001) and obesity (Control group:4,9, Intervention group 51,63 p < 0,001) along with a positive attitude score towards fighting obesity (Control group: 1,8, Intervention group 13,6 p < 0,001). Still, the overall change was more remarkable for the intervention group, as indicated by the significant interaction effect of the analysis. Anxiety was decreased only in the intervention group (Control group:0,11, Intervention group − 0,17 p < 0,005). Analysis for QOL during follow-up showed that Physical Health and Level of Independence was improved in both study groups but the degree of improvement was more significant in the intervention group (Control group 0,31,Intervention group 0,73 p < 0,001). Psychological Health was improved only in the intervention group, with better scores at 6 and 12 months compared to controls (Control group 0,28,Intervention group 1,42 p < 0,001). Furthermore, Social relationships were improved only in the intervention group (Control group 0,02, Intervention group 0,56 p < 0,001).

**Conclusions:**

The results of the present study showed that the participants in the intervention group showed significant improvement in knowledge, attitudes, and beliefs after using the internet as a learning tool. The intervention group also showed significantly reduced anxiety and depression arising from chronic illness. All of this resulted in an improved quality of life regarding physical Health, mental Health, and social relationships. Technology and online-based health promotion programs can revolutionize how we approach the prevention and management of chronic and terminal illnesses by improving accessibility, personalizing care, increasing engagement and motivation, improving data analysis, and disease management.

## Introduction

The development of the World Wide Web and the Information and Communication Technologies in the 1990s helped spread the Internet from one side of the globe to the other. This enabled people to develop applications, to directly access a wide range of information, and to communicate more easily [1].

It is a fact that the number of new users is constantly growing. In fact, in 2020, perhaps due to the pandemic, there was a 60% increase in the global digital population. In Greece in the same year, there was a 73% increase in internet home access [2]. The world wide web is now considered an effective platform that can be used in the context of trials for the dissemination of public health through online interventions. The tools it provides can help in educating individuals about health issues who live in remote areas with limited access to health, allowing them to learn and receive higher quality education compared to where they reside. Any barriers to communication for the prevention and promotion of a healthy lifestyle are now overcome by the intervention of the internet.[3].

Smart devices (smartphones and tablets) provide easy and daily access to information, communication and opportunities for motivation. Social networking platforms have invaded the everyday life of users with more than 3 billion monthly active users. And with percentages that still reach up to 70% of adults using at least one platform for 2 h daily [4].

Thousands of health-related websites and applications have been developed, enabling new, friendly health education programs to be implemented [5, 6]. In the 70s, the first health education program focused on changing the behavior of individuals through transmission of knowledge to specific groups. In the 80s, the programs focused on group training methods and printed materials, as well as media campaigns. In the 90s, with the rapid development of digital technology, CDROM applications were included in the health education programs [7]. Finally, in the new millennium and the internet era, interactive web applications now play a major role in health education, increasing their effectiveness in target populations [8, 9]. Social media are now considered a popular tool in communication and education, and the possibilities they provide for modifying health behaviors are significantly increased through the developed programs. For example, research results regarding the impact of social media on promoting health showed that Instagram and Twitter were a medium that concerned user behavior changes, while through YouTube and Facebook, users recorded increased behavior change interventions [10]. Health education is most beneficial in chronic diseases such as diabetes and morbid obesity, which can be positively affected by changes in attitudes, beliefs, and lifestyle (behavior modification) [11–13]. For example, patients with increased knowledge, self-care behaviors, and specific attitudes and beliefs result in better control of blood sugar levels among individuals with type 2 diabetes [14].

According to the World Health Organization (WHO), trends on both diabetes and obesity are not encouraging. Diabetes is considered one of the top ten causes of death. As defined by the American Diabetes Association, diabetes type 2 is a chronic medical condition characterized by high glucose levels due to insulin resistance and/or inadequate insulin production by the pancreas. It is the most common form of diabetes, and typically develops in adulthood [15]. The rates for 2019 are estimated at approximately 463 million cases of diabetes worldwide in the 20–79 age group. By 2045 we expect a 51% increase, with the number of people with diabetes reaching about 700 million. In Europe, it is estimated that in 2021 the number of diabetes cases was 540 million. Among all cases of diabetes, more than 90% are type 2 diabetes (T2D). The annual progression from prediabetes to T2D ranges from 5 to 10% and one of the most significant predisposing factors in the appearance of type 2 diabetes is obesity [16]. It is estimated that obese individuals have a 3–4 times higher frequency of developing T2D compared to individuals with a normal BMI [17].

As far as obesity is concerned, as defined by WHO is an excessive accumulation of body fat, leading to a significant increase in body weight that may affect an individual’s health. The World Health Organization (WHO) defines obesity as a body mass index (BMI) of 30 or higher [18]. In 2016, about 2 billion adults were overweight or obese [19] while approximately 41 million children under the age of 5 and 340 million children between 5 and 18, especially in the developed world, are considered overweight or obese. Since there is a strong link between obesity and diabetes, having so many children with obesity can only lead to increased incidence of diabetes in the future. So addressing obesity now can prevent diabetes at the future.

The high prevalence of these metabolic disorders, their impact on public health, and the increasingly rising social and economic cost are pushing us towards finding innovative interventions through the internet, guided by the therapeutic team (doctor, dietitian, psychologist, nurse, psychiatrist). The aim of this study was to develop a modern Health Promotion model, through continuing education and participation using interactive online applications that follow the modern technology, is specifically designed to respond individually and personalized to the educational needs of each patient regardless of the education level, since the “wall” of each patient was unique and created according to their needs. The goal was to positively impact knowledge, behavior and eventually quality of life for those who suffer from obesity or diabetes type2. It has been proven that education for patients with type 2 diabetes and obesity is positively related to their adherence to treatment. In addition, providing education on disease management, nutrition, physical exercise, and complication prevention can help patients change their lifestyles and improve their health.

Furthermore, the increase in rates of type 2 diabetes and obesity in Greece and worldwide is a serious public health issue. Therefore, a health education program that focuses on preventing and managing these diseases can help reduce their impact on population health and improve the quality of life for patients. Developing health education programs for patients with type 2 diabetes and obesity is essential to improving public health services.

## Methods

This is a prospective interventional study on patients with obesity or type 2 diabetes age group of 18–60 years old, where it was conducted during 2019–2020 in Endocrine Unit, Second Propaedeutic Department of Internal Medicine, Attikon University Hospital, National and Kapodistrian University of Athens, Athens, Greece.

Patients were recruited at the Endocrine Unit of the Ddepartment. All the patients who met the eligibility criteria were distributed randomly into 2 groups (control and intervention). Possession of a smart phone and internet access was essential for their inclusion in the protocol. Patients with severe mental diseases and patients with severe commodities like cancer, or renal failure were excluded from the study. The randomization was based on the order of the appointments, with the odd numbers being assigned to the control group and the evens to the intervention group.

The measurements of the individuals who participated in the study included the calculation of BMI, current and mean waist circumference, glycosylated hemoglobin, blood pressure, cholesterol, and questionnaires. All the participants were given questionaries concerning quality of life [20], anxiety and depression (HADS) [21] attitudes and beliefs [22], knowledge about their condition [23], and other general questions to establish a baseline (demographic characteristics, internet use, everyday activities, etc.). In the control group the questionnaires were given in person, while in the intervention group they were sent electronically. Participants in both groups completed the same questionnaires at the time of recruitment, at 6 months, and again at 12 months. All the data collection performed from the principal investigator to decries the risk of bias. All patients sign an informed consent form before being included in the research groups to participate and publish the data. The consent form was reviewed and approved from the ethic committee of the hospital.

For the participants in the control group, a traditional health promotion model was followed. Printed materials were sent every 3 months, and in small groups 4 lectures was given at 6th months of the intervention. The clinic’s medical staff performed the lectures, which were approximately 45 min long and included general epidemiological information about diabetes and obesity and the importance of a healthy lifestyle. So the overall implication of the control group participants was calculated to be approximately 24 h a year for both synchronous (lectures) and asynchronous activities (study material).

For participants in the intervention group, a health promotion program was created according to the goals of the research. For the purposes of the study a website was developed called www.ipromotehealth.gr, in order to provide individualized training where participants created their username and password to access the platform.

The website included two knowledge games: ***“i play 4 health”*** and **“i play 2 learn”**. The first game was simply answering questions, while the second one added a competitive element among the other participants.

According to the instructions given by the researcher to the intervention group, each patient was required to dedicate at least 15 min twice a week, with a minimum interval of 2 days, to complete the 2 games that were developed (each consisting of 10 questions) as part of the study, and to read the texts posted on the website. The purpose of the games was to provide personalized information to each patient, as incorrect answers in the games would contribute to the creation of a corresponding part of the “wall”. The texts were written by the research team and clinical staff, and the average reading time for each text did not exceed 3 min. In addition to these texts, general information promoting healthy eating, exercise, etc., as well as epidemiological data, were posted on the “wall”. The total engagement of participants in the intervention was approximately 25 h per year.

Compliance of participants was monitored weekly through Google Analytics. In case of non-compliance, the principal investigator would send a reminder email or SMS. Access to the specially designed platform was available exclusively to each participant for 12 months and could be used at any time, according to their own preference.

In particular, the home page of each participant included:

1. General articles that were common to the whole group, about a healthy lifestyle, smoking, the importance of physical activity, etc.
2. Customized health information based on the incorrect answers from the games.
3. Personalized tips that emerged from the attitudes and beliefs section.

This research protocol was approved by the ethics committee of the University General Hospital “Attikon”, and written consent of the participants was obtained.

### Statistical analysis

The analysis of the data was per protocol. Quantitative variables were expressed as mean (Standard Deviation) or as median (interquartile range). Qualitative variables were expressed as absolute and relative frequencies. Independent samples Student’s t-tests were used for the comparison of mean values between the two groups. For the comparison of proportions chi-square and Fisher’s exact tests were used. Repeated measurements analysis of variance (ANOVA) was adopted to evaluate the changes observed in knowledge scores on diabetes and obesity, attitude score towards fighting obesity, quality life scores, anxiety and depression scales among the two groups over the follow up period. Log transformations were made in case of not normal distribution. Power analysis methodology represented a design, with two groups of the between-subject factor of the studied groups and three levels of the within-subjects factor of time. For this design, 72 participants (36 per group) achieved a power of 0.95 for the between-subjects main effect at an effect size of 0.35; a power of 0.95 for the within-subjects main effect at an effect size of 0.20; and a power of 0.95 for the interaction effect at an effect size of 0.20. All reported p values are two-tailed. Statistical significance was set at p < 0.05 and analyses were conducted using SPSS statistical software (version 22.0).

## Results

Sample consisted of 72 patients (36 in the control group and 36 in the intervention group). Demographics and other characteristics of the two study groups are presented in Table 1. The mean age was 47.8 years (SD = 12.2 years) for the control group and 42.7 years (SD = 13.5 years) for the intervention group (p = 0.293). The two groups of participants were also similar in terms of sex, educational status, health status and other characteristics resented in Table 1. Table 2 shows the changes over the follow up period for in knowledge and attitudes for the control and intervention group.

Both study groups had a significant increase in knowledge score on diabetes and obesity along with positive attitude score towards fighting obesity, but the overall change was greater for the intervention group as indicated from the significant interaction effect of the analysis. At both 6 and 12 months the intervention group had better performance concerning in knowledge scores and positive attitude score towards fighting obesity, as compared to control group.

Changes in anxiety and depression during follow up for the two study groups are shown in Table 3. Anxiety was decreased only in the intervention group, while depression was decreased in both groups.

Analysis for QOL during follow up (Table 4) showed that Physical health and Level of Independence was improved in both study groups but the degree of improvement was greater in the intervention group. Psychological Health and Spirituality was improved only in the intervention group, which had better scores at both 6 and 12 months in comparison to controls. Furthermore, Social relationships were improved only in the intervention group.

**Table 1:** Sample characteristics

	Control group (N = 36; 50%)	Intervention group (N = 36; 50%)	
	N (%)	N (%)	Ρ
Gender			
Men	8 (22.2)	12 (33.3)	0.293+
Women	28 (77.8)	24 (66.7)	
Age, mean (SD)	47.8 (12,2)	42.7 (13.5)	0.249‡
Educational level			
High school	6 (16.7)	3 (8.3)	0.501++
2-year college	2 (5.6)	6 (16.7)	
Technical University	12 (33.3)	9 (25.0)	
University	10 (27.8)	11 (30.6)	
MSc	6 (16.7)	7 (19.4)	
Family status			
Unmarried	14 (38.9)	14 (38.9)	0.902++
Married	15 (41.7)	15 (41.7)	
Separated	1 (2.8)	2 (5.6)	
Divorced	6 (16.7)	4 (11.1)	
Widowed	0 (0.0)	1 (2.8)	
Children			
No	14 (38.9)	16 (44.4)	0.633+
Yes	22 (61.1)	20 (55.6)	
Living conditions			
Alone	11 (30.6)	12 (33.3)	0.800+
With others	25 (69.4)	24 (66.7)	
Occupation			
Unemployed	16 (44.4)	18 (50.0)	0.777+
In public sector	7 (19.4)	5 (13.9)	
In private sector	7 (19.4)	9 (25.0)	
Free lancers	6 (16.7)	4 (11.1)	
Place o f residence			
Attica	30 (83.3)	32 (88.9)	0.496+
Out of Attica	6 (16.7)	4 (11.1)	
Health status			
Verry poor	0 (0.0)	0 (0.0)	0.627++
Poor	0 (0.0)	0 (0.0)	
Not poor nor good	13 (36.1)	9 (25.0)	
Good	20 (55.6)	24 (66.7)	
Very good	3 (8.3)	3 (8.3)	

^+^Pearson’s chi-square test; ^++^Fisher’s exact test; ^‡^Student’s t-test

**Table 2:** Changes in knowledge and attitudes during follow up for the two study groups

		Baseline		6 months		12 months		Change		
		Mean (SD)	Median (IQR)	Mean (SD)	Median (IQR)	Mean (SD)	Median (IQR)	Mean (SD)	**P** ^**2**^	**P** ^**3**^
Knowledge score on diabetes	Control group	13.65 (5.97)	13 (9 ─ 20)	21.71 (2.28)	22 (20 ─ 24)	16.88 (5.07)	18 (13 ─ 22)	3.24 (4.18)	**< 0.001**	**< 0.001**
	Intervention group	12.06 (2.28)	12 (11 ─ 14)	23.59 (1.7)	24 (24 ─ 24)	23.94 (0.24)	24 (24 ─ 24)	11.88 (2.32)	**< 0.001**	
	P^1^	0.782		**0.015**		**< 0.001**				
Knowledge score on obesity	Control group	58.01 (24.8)	64.71 (35.29 ─ 82.35)	80.07 (8.92)	82.35 (76.47 ─ 88.24)	62.91 (22.14)	67.65 (47.06 ─ 82.35)	4.9 (11.72)	**< 0.001**	**< 0.001**
	Intervention group	36.27 (10.48)	35.29 (29.41 ─ 41.18)	86.76 (2.58)	88.24 (85.29 ─ 88.24)	87.91 (1.37)	88.24 (88.24 ─ 88.24)	51.63 (10.72)	**< 0.001**	
	P^1^	**< 0.001**		**< 0.001**		**< 0.001**				
Positive attitude score towards fighting obesity	Control group	61.3 (8.9)	65 (55 ─ 70)	70.7 (8.6)	75 (65 ─ 75)	63.1 (9)	65 (60 ─ 70)	1.8 (3)	**< 0.001**	**0.001**
	Intervention group	66.3 (10.9)	67.5 (60 ─ 75)	78.6 (8.4)	80 (70 ─ 85)	79.9 (8.1)	80 (75 ─ 90)	13.6 (16)	**< 0.001**	
	P^1^	0.061		**< 0.001**		**< 0.001**				

*Note.* Analysis was conducted with logarithmic transformations

^1^p-value for group effect; ^2^p-value for time effect; ^3^Effects reported include differences between the groups in the degree of change (repeated measurements ANOVA)

**Table 3:** Changes in anxiety and depression during follow up for the two study groups

		Baseline		6 months		12 months		Change		
		Mean (SD)	Median (IQR)	Mean (SD)	Median (IQR)	Mean (SD)	Median (IQR)	Mean (SD)	**P** ^**2**^	**P** ^**3**^
Anxiety	Control group	4.61 (3.05)	6 (1 ─ 7)	4.67 (3.07)	6 (1 ─ 7)	4.72 (3.06)	6 (1 ─ 7)	0.11 (0.46)	0.942	0.149
	Intervention group	3.81 (3.47)	4 (0 ─ 7)	3.33 (3.47)	2.5 (0 ─ 7)	3.64 (3.49)	4 (0 ─ 7)	-0.17 (1.7)	**0.005**	
	P^1^	0.225		0.062		0.120				
Depression	Control group	5.22 (2.84)	6 (2.5 ─ 7)	4.94 (2.99)	6 (2.5 ─ 7)	5 (3.5)	6 (1.5 ─ 7)	-0.22 (1.29)	**0.043**	0.191
	Intervention group	3.97 (2.89)	4.5 (2 ─ 6)	3.39 (2.76)	3.5 (0.5 ─ 6)	3.78 (3.09)	3.5 (0.5 ─ 7)	-0.19 (2.03)	**0.027**	
	P^1^	**0.029**		**0.022**		0.274				

*Note.* Analysis was conducted with logarithmic transformations

^1^p-value for group effect; ^2^p-value for time effect; ^3^Effects reported include differences between the groups in the degree of change (repeated measurements ANOVA)

**Table 4:** Changes in QOL during follow up for the two study groups

		Baseline	6 months	12 months	Change		
		Mean (SD)	Mean (SD)	Mean (SD)	Mean (SD)	P^2^	P^3^
Overall QoL/ health facet	Control group	14.39 (2.43)	14.39 (2.43)	14.39 (2.43)	0.00 (0.00)	1.000	0.236
	Intervention group	14.56 (2.01)	14.78 (1.93)	14.72 (1.92)	0.17 (0.74)	0.099	
	P^1^	0.752	0.454	0.521			
Physical health and Level of Independence	Control group	13.62 (1.79)	13.8 (1.93)	13.93 (2.04)	0.31 (0.46)	**0.001**	**0.001**
	Intervention group	14.06 (1.68)	14.59 (1.66)	14.79 (1.77)	0.73 (0.5)	**< 0.001**	
	P^1^	0.252	0.054	0.060			
Psychological Health and Spirituality	Control group	13.43 (1.86)	13.57 (1.93)	13.7 (2.01)	0.28 (0.56)	0.072	**< 0.001**
	Intervention group	13.54 (1.58)	14.59 (1.5)	14.95 (1.61)	1.42 (0.94)	**< 0.001**	
	P^1^	0.777	**0.013**	**0.005**			
Social relationships	Control group	14.29 (2.4)	14.29 (2.4)	14.31 (2.4)	0.02 (0.3)	0.962	**0.003**
	Intervention group	14.13 (2.56)	14.64 (2.23)	14.7 (2.26)	0.56 (0.79)	**< 0.001**	
	P^1^	0.814	0.489	0.488			
Environment	Control group	11.97 (1.43)	11.96 (1.47)	12.03 (1.34)	0.06 (0.12)	0.260	0.510
	Intervention group	12.39 (1.65)	12.51 (1.65)	12.39 (1.69)	0 (0.08)	0.250	
	P^1^	0.238	0.202	0.223			

^1^p-value for group effect; ^2^p-value for time effect; ^3^Effects reported include differences between the groups in the degree of change (repeated measurements ANOVA)

## Discussion

Education as a public health service is of vital importance. Specifically when prevention and treatment are part of a strategy to control chronic conditions such as type 2 diabetes or obesity. Therapeutic patient education [WHO] is considered of primary importance so that patients can first understand the nature of their disease and then be empowered with knowledge and skills to manage their symptoms, especially in chronic conditions [24].

Health promotion interventions are considered effective in preventing and improving the overall health of the target population. However, their range could be made more effective through internet-based programs from authorized agencies, taking into account users’ actual needs and expectations [3]. A large number of health-related applications focusing on prevention and treatment are available today. These educational tools are aimed mainly at younger age groups, having a greater impact than traditional techniques because younger generations are daily users of internet and these types of interventions are more friendly to them [25]. The use of such educational tools has been growing for the last 20 years, but in the last 5 years this growth has accelerated due to the wider use of smart devices [26]. Unfortunately most of them are programs mainly focusing on diet and exercise, neglecting the mental impact of these conditions on overall health.

Evidence for the impact of these applications on users’ lifestyles is compelling, according to a 2019 Xinghan et al. meta-analysis of more than 23 studies [27].

These applications could improve health promotion by facilitating interdisciplinary collaboration, better doctor and patient communication, exchange of ideas between patients and targeted information [28]. This is in contrast to health promotion programs that have focused mainly on passive, lecture-based models without opportunities for interaction [29].

The aim of this study was to provide targeted and personalized information to participants according to their knowledge deficiencies. Participants answered a series of questions through interactive games and depending on the wrong answers they received the corresponding information. Using this model, a significant improvement in their knowledge scales was recorded compared to the traditional model of health promotion used in the control group. In Garrett et al. published a randomized study in people with diabetes using a **learning map** for continuous training. It showed that educating, and changing attitudes and beliefs in the intervention group led to better outcomes, compared to the control group that had the classical educational approach [30]. Active, continuous and interactive participation motivates the target individuals towards a more positive attitudes about their condition [31].

The importance of the interactive educational process that takes place through these applications is great and is highlighted by research such as a systematic review of Norris et al., in 2001. This study focused on diabetes self-management education through patients collaborating with others in the same group [32]. In contrast, there are interventions of health education programs that focus mainly on passive models without interaction between the participants and are simply done in the form of a lecture [33]. A prospective study, in weight management clinics only gave patients diet programs and behavioral interventions seeking to change attitudes toward obesity. After 12-month the subjects’ knowledge remained low without significant improvement [34].

The development of various applications that are used daily by millions of users enables direct communication between people of different age groups but who have something in common, such as a health condition. Indeed, a meta-analysis by Laranjo et al. in 2015 showed that, with widespread social media such as Twitter and Facebook, the effect on behavior modification was greater since these networks make it easier to communicate in private groups [35]. Greater knowledge seems to significantly affect the positive attitudes and optimism of chronic patients and especially patients with diabetes and obesity [36]. This seems to be the case because the determining factor that influences positive outcomes for those with metabolic diseases is lifestyle change. This factor is exactly what health education programs seek to address [37]. Although the importance of optimism has been widely studied in the health of chronic patients, there is limited information on the factors that determine this positivity [38].

Positive attitudes towards chronic illnesses can help to alleviate anxiety and depression, which are major factors influencing quality of life. [39] A study by Luppino et al. in 2010, revealed the vicious cycle that can occur between depression and obesity [40]. In the present study the participants in the intervention group showed significantly reduced levels of anxiety and depression after 6 months compared to the participants in the control group. Similarly, another study involving 76 people with both morbid obesity and depression showed the effectiveness of depression treatment that also included a health promotion program for weight control. Patients had positive results in terms of weight loss and improved optimism [41].

Providing information and encouraging optimism in patients with chronic diseases, such as diabetes, also helps to change their attitudes and beliefs and consequently to improve the compliance with the treatment. In giving patients more control over their treatment, we can reduce potential complications from the disease [42]. In the present study, more positive attitudes and beliefs towards their disease were recorded compared to the control group. Indeed, other studies come to this conclusion also.

In 2004, Wassenberg et al. showed that positive attitudes in people with hypertension towards their chronic disease resulted in better regulation of the disease, as participants had more consistent monitoring of blood pressure [43]. In addition, a study using a web-based online games approach to changing attitudes and beliefs showed that participants in the intervention group had better HbA1c levels after 3 months of intervention, due to lifestyle changes encouraged in the program. Patients reported they felt more secure in a web base self-management education health environment, compared to the traditional model [44].

Another study by Baranowski et al. in 2016, in collaboration with many organizations, identified the most beneficial features for health education games: that they be interactive, have feedback, allow for agency and control, identity, and immersion [45].

In the present research these elements were included in the development of the two online knowledge games. Greater knowledge resulting in behavior modification was observed since the participants received direct information about their performance (score) as well as customized information depending on their incorrect answers. Indeed, the value of this approach can also be seen in publications such as a study by Espinosa-Curiel et al. in 2020, which developed an online knowledge game aimed at changing children’s eating habits. In just 6 weeks there was an improvement in the level of knowledge as well as a more positive attitude towards a healthier diet. The children’s parents agreed that the game had a positive effect on them as they could now recognize more than 10 unhealthy foods [46].

Like most studies performed with questionaries, the present study has some limitations that should be considered. Apart from the limited sample size, limited depth of information, and possible misunderstandings or interpretation errors from the participants, mainly due to language barriers. The main limitation is the self-report bias. Questionnaires rely on self-reported data, and sometimes the participants may not always be honest or accurate in their responses. In addition, they may be influenced by social desirability bias or other factors affecting their answers, so researchers have limited control over variables that could affect the results. While questionnaires can provide valuable insights into participants’ knowledge, attitudes, and beliefs, they are not without limitations, and their findings should always be considered in the context of their limitations.

## Conclusion

It appears that the study was conducted during the COVID-19 pandemic, which posed significant challenges due to government restrictions. Diabetes was one of the comorbidities with increased incidence in COVID-19 patients as the virus is related to glucose dysregulation or hyperglycemic hyperosmotic state [47]. Additionally, many health promotion programs focusing on type 2 diabetes and obesity had been canceled. The development of patients-based online health promotion programs (such ipromotehealth.gr) is significant for patients as they continuously receive support to control their glycemic control.

The present intervention was a challenge for the participants as they were unfamiliar with such programs. However, the personalized approach, the online material, and the monitoring from the research team through Google Analytics, delivered targeted feedback and encouraged participation in a healthier lifestyle, better monitoring of glucose levels, etc. The final analysis of the research results showed that the participants in the intervention group showed significant improvement in knowledge, attitudes, and beliefs after using the internet as a learning tool, particularly compared to the control group which used a traditional health promotion approach. The intervention group also showed a significant reduction of anxiety and depression arising from chronic illness. All of this resulted in an improved quality of life in regards to physical health, mental health, and social relationships. Those involved in health education and promotion not only must integrate modern tools into their existing programs, but also must design new interventions using the philosophy of the internet and social media, through the growing potentials of smart phones. Using these technologies, and looking into the future with the artificial intelligence, we can allow users to benefit from both traditional and experiential methods. Practically, the internet allows health promotion providers to easily and quickly customize health education to fit the individual need.

Technology and online-based health promotion programs have the potential to play a significant role in addressing a wide range of chronic and terminal illnesses. Some potential future implications are improved accessibility by allowing patients to access information and support from their homes. This can be particularly important for patients who have difficulty traveling or live in rural or remote areas. Another significant implication is the personalization of care. Technology can help health promotion programs better tailor their care to individual patients, considering factors such as age, gender, health status, and lifestyle. This can help to make care more effective and engaging for patients and increase motivation for example development of games that can also be used to make health promotion programs more engaging and fun. Also, online programs are cost-effective by reducing the need for face-to-face consultations and other traditional healthcare services.

In conclusion, technology and online-based health promotion programs have the potential to revolutionize the way we approach the prevention and management of chronic and terminal illnesses. By improving accessibility, personalizing care, increasing engagement and motivation, improving data analysis, and improving disease management, these programs can help to improve health outcomes and reduce the burden of illness on individuals and society.

## Data Availability

The datasets used and analysed during the current study are available from the corresponding author upon reasonable request.
